# Production of Indigo by Recombinant *Escherichia coli* with Expression of Monooxygenase, Tryptophanase, and Molecular Chaperone

**DOI:** 10.3390/foods11142117

**Published:** 2022-07-16

**Authors:** Lingyan Du, Jianming Yue, Yiying Zhu, Sheng Yin

**Affiliations:** 1 School of Food and Health, Beijing Technology & Business University, Beijing 100048, China; lingyan.du@genetronhealth.com (L.D.); yjm2513@163.com (J.Y.); 1903010116@st.btbu.edu.cn (Y.Z.); 2 Beijing Advanced Innovation Center for Food Nutrition and Human Health, Beijing Technology & Business University, Beijing 100048, China; 3 Beijing Engineering and Technology Research Center of Food Additives, Beijing Technology & Business University, Beijing 100048, China

**Keywords:** indigo, monooxygenase, tryptophanase, molecular chaperone, *Escherichia coli*

## Abstract

Indigo is an important pigment widely used in industries of food, cosmetics, and textile. In this work, the styrene monooxygenase StyAB from *Pseudomonas putida* was co-expressed with the tryptophanase TnaA and the chaperone groES-groEL in *Escherichia coli* for indigo production. Over-expression of the gene *styAB* endowed the recombinant *E. coli* AB with the capacity of indigo biosynthesis from indole and tryptophan. Tryptophan fermentation in *E. coli* AB generated about five times more indigo than that from indole, and the maximum 530 mg/L of indigo was obtained from 1.2 mg/mL of tryptophan. The gene *TnaA* was then co-expressed with *styAB*, and the tryptophanase activity significantly increased in the recombinant *E. coli* ABT. However, *TnaA* expression led to a decrease in the activity of StyAB and indigo yield in *E. coli* ABT. Furthermore, the plasmid pGro7 harboring groES-groEL was introduced into *E. coli* AB, which obviously promoted the activity of StyAB and accelerated indigo biosynthesis in the recombinant *E. coli* ABP. In addition, the maximum yield of indigo was further increased to 550 mg/L from 1.2 mg/mL of tryptophan in *E. coli* ABP. The genetic manipulation strategy proposed in this work could provide new insights into construction of indigo biosynthesis cell factory for industrial production.

## 1. Introduction

Indigo is one of the oldest pigments used by human beings with a history of thousands of years, and it is still widely used in food, pharmaceuticals, cosmetics, and textile industries [1,2]. Indigo production is mainly achieved by extraction from plants and chemical synthesis [3]. However, indigo preparation from plants has obvious disadvantages in cost and yield, and chemical synthesis inevitably brings about harmfulness to human health and the environment due to toxic compounds from the reaction systems [4]. Previous studies reported indigo could be produced by various microorganisms such as *Pseudomonas* and *Acinetobacter* [5,6,7,8,9,10], which provides economic, effective, and eco-friendly approaches for indigo production. Various indigo biosynthesis pathways were found in different microbial species. Generally, indigo is generated via oxidation of the substrate by catalysis of oxygenase. In *Pseudomonas* species, multiple oxygenases, including xylene oxygenase, toluene-4-monooxygenase, toluene dioxygenase, 2-naphthoic acid oxygenase, naphthalene dioxygenase, and styrene monooxygenase, were proved to express the catalytic activities of indigo biosynthesis [8,11]. O’Connor et al. (1997) proposed the pathway of indigo biosynthesis from indole in *Pseudomonas*. Briefly, indole is oxidized into indole oxide by monooxygenase; indole oxide is then transformed to indoxyl by isomerase; and two indoxyl molecules form indigo by dimerization [12]. However, not only indigo but also the structurally similar by-product indirubin could be generated via the alternative branch pathway by catalysis of dioxygenase [13,14]. Therefore, indole conversion by catalysis of monooxygenase is a shortcut to obtain pure indigo.

In our previous work, over-expression of the styrene monooxygenase gene *styAB* was conducted in *Pseudomonas putida*, and it significantly enhanced indigo production from indole, revealing that the monooxygenase StyAB acted as the key rate-limiting enzyme for indigo biosynthesis [15]. In this work, the styrene monooxygenase gene *styAB* from *P. putida* was heterologously expressed in *Escherichia coli* for highly efficient indigo production, and co-expression of *styAB* with the tryptophanase gene *TnaA* and the molecular chaperone *groES-groEL* was further performed in *E. coli* to construct an indigo production system from tryptophan. The genetic manipulation strategy proposed in this work provided new insights into construction of indigo biosynthesis cell factory for industrial application.

## 2. Materials and Methods

### 2.1. Strains, Plasmids and Culture Conditions

Strains and plasmids used in this work are listed in Table 1. *E. coli* strains were grown in Luria–Bertani (LB) medium at 37 °C with vigorous shaking. *P. putida* strain was cultured at 30 °C in LB medium with vigorous shaking. When needed, kanamycin (50 μg/mL) and chloramphenicol (35 μg/mL) were added into LB medium for *E. coli* strain screening.

### 2.2. DNA Manipulation Techniques

Standard DNA manipulation techniques were performed as described by Green and Sambrook [16]. Bacterial genomic DNA was prepared using the TIANamp Bacteria DNA Kit (TIANGEN, Beijing, China) following the manufacturer’s instructions. Plasmid DNA from *E. coli* was prepared using the High-purity Plasmid Miniprep Kit (TIANGEN, Beijing, China) according to the manufacturer’s instructions. DNA amplification was performed using the TakaRa Primer SRTAR MAX DNA Polymerase following the manufacturer’s protocol (Takara, Beijing, China). Restriction endonuclease digestion and DNA ligation were conducted according to the manufacturer’s instructions (Takara, Beijing, China). Standard heat-shock transformation method was used to introduce plasmid DNA to *E. coli* [16]. Total mRNA from *E. coli* was prepared using the Trizol Extraction Kit according to the manufacturer’s instructions (BioTeke, Beijing, China). RNA was subject to reverse transcription to generate cDNA using FastKing RT Kit (TIANGEN, Beijing, China) following the manufacturer’s protocol. Quantitative Real-Time PCR (q RT-PCR) was performed using the SuperReal PreMix Plus (SYBR Green) Kit (TIANGEN, Beijing, China) in Light Cycler Nano System (Roche Diagnostics, Indianapolis, IN, USA) with the following cycling conditions: 95 °C for 10 s, followed by 45 cycles of 95 °C for 10 s, 56 °C for 30 s, and 72 °C for 45 s. The 16S rRNA gene was used for transcript normalization. All reactions were performed in triplicate. Data were analyzed using the 2^−ΔΔCt^ method corrected for primer efficiencies using the untreated group mean as the reference condition [17]. Primers used in this work were listed in Table 2.

### 2.3. Vectors Construction for Gene Expression in E. coli

The *styAB* gene was amplified by PCR from the genomic DNA of *P. putida* B4 using the specific primers styAB-F and styAB-R designed according to the sequence of styrene monooxygenase gene (GenBank accession no. DQ177365.1) from *P. putida*. The amplicon of *styAB* was inserted into the multiple cloning site (MCS) of the *E. coli* expression vector pBK-CMV to construct the recombinant vector pBK-AB (Figure S1), which was transformed into *E. coli* DH5α, and the recombinant strain *E. coli* AB was screened on LB agar plates containing kanamycin.

The *TnaA* gene was amplified by PCR from the genomic DNA of *E. coli* DH5α using the specific primers TnaA-F and TnaA-R designed according to the sequence of tryptophanase gene (GenBank accession no. NC_000913.3) from *E. coli*. The amplicon of *TnaA* was inserted into the upstream site of *styAB* in MCS of pBK-AB to construct the recombinant vector pBK-ABT (Figure S1). pBK-ABT was transformed into *E. coli* DH5α to construct the recombinant strain *E. coli* ABT.

The chaperone plasmid pGro7 was transformed into *E. coli* AB and *E. coli* ABT, respectively, and the recombinant strains *E. coli* ABP and *E. coli* ABTP were screened on LB agar plates containing kanamycin and chloramphenicol. The recombinant vectors were verified by sequencing and alignment analysis using DNAMAN software package and BLAST Program at NCBI against the GenBank database, and the recombinant strains were verified by plasmid profile and sequencing.

### 2.4. Indigo Production by E. coli Fermentation

For indigo production, fresh overnight culture of *E. coli* was inoculated (1%) in the fermentation medium (17 g/L Na_2_HPO_4_ • 12 H_2_O, 3 g/L KH_2_PO_4_, 1 g/L NH_4_Cl, 0.5 g/L NaCl, 0.1 g/L MgSO_4_, and 3 g/L yeast extract) containing indole or tryptophan as the substrates, and fermentation was conducted at 30 °C with vigorous shaking at 200 rpm for 24~48 h. For fermentation of the *E. coli* strain harboring the chaperone plasmid pGro7, fresh overnight culture of *E. coli* was inoculated (1%) in LB medium and cultured at 37 °C with vigorous shaking at 200 rpm. When cells density reached 0.2 (OD 600 nm), 150 μg/mL of arabinose was added in LB medium, and cells were cultured at 30 °C with vigorous shaking at 200 rpm until OD 600 nm reached 0.8. The fresh culture was then inoculated (1%) in the fermentation medium, and fermentation was conducted as described above. The fermentation data were representative of three independent experiments performed in triplicate.

### 2.5. Enzymatic Activity Assay

Monooxygenase activity was measured by indole consumption as described previously [15]. Cells were harvested from cultures by centrifugation at 10,000× *g* for 10 min, washed with potassium phosphate buffer (50 mM, pH 7.0), and resuspended in the same buffer containing 5 mM indole. Cell suspension was incubated in shaking water bath at 30 °C at 150 rpm for 30 min. Indole depletion was determined by HPLC, and 1 unit (U) of monooxygenase activity was defined as 1 μM indole depletion in 30 min.

Tryptophanase activity was assayed by indole production from tryptophan. Cells were harvested from cultures by centrifugation at 10,000× *g* for 10 min, washed with phosphate buffer (100 mM, pH 7.0), and resuspended in the same buffer. Cell resuspension solution was subject to sonication in ice-bath, and the supernatant was collected by centrifugation at 10,000× *g* for 5 min. Cell supernatant was mixed with glutathione (5 mM) and tryptophan (5.0 mg/mL) and incubated in shaking water bath at 37 °C at 150 rpm for 10 min. Indole production was determined by HPLC, and 1 unit (U) of tryptophanase activity was defined as 0.01 μm indole production in 10 min. The results were representative of three independent experiments performed in triplicate. Significant differences between different strains were identified by the unpaired Student’s *t*-test or ANOVA analysis.

### 2.6. Measurement of Indole, Indigo, and Tryptophan

For indigo determination, fermentation culture was centrifuged at 10,000× *g* for 10 min to collect blue indigo pellets, which were washed with water and resuspended in dimethyl formamide (DMF). The indigo suspension was subject to sonication for 5 min repeatedly and filtrated with 0.22 μm millipore for HPLC analysis. Indigo and indole were measured by a HPLC system (Agilent 1290, Agilent, Santa Clara, CA, USA) equipped with an Agilent Eclipse plus C18 RRHD column (1.8 µm, 2.1 mm × 50 mm) and diode array detector [15]. The mobile phase was water/methanol (10: 90, *v*/*v*), and the operating conditions were as follows: detection at 610 nm and flow rate of 0.2 mL/min.

For tryptophan determination, fermentation culture was centrifuged at 10,000× *g* for 5 min to collect supernatant. The supernatant was filtrated with 0.22 μm millipore and used for tryptophan determination by a HPLC system (Agilent 1200, Agilent, Santa Clara, CA, USA) equipped with an Agilent C18 column (5.0 µm, 150 mm × 4.6 mm) and diode array detector. The mobile phase was 0.03% KH_2_PO_4_ solution/methanol (90:10, *v*/*v*), and the operating conditions were as follows: detection at 278 nm and flow rate of 1.0 mL/min. The results were representative of three independent experiments performed in triplicate. Significant differences between different strains were identified by the unpaired Student’s *t*-test.

## 3. Results

### 3.1. Expression of Styrene Monooxygenase Gene StyAB Generated Indigo Biosynthesis in E. coli

**Figure 1:**
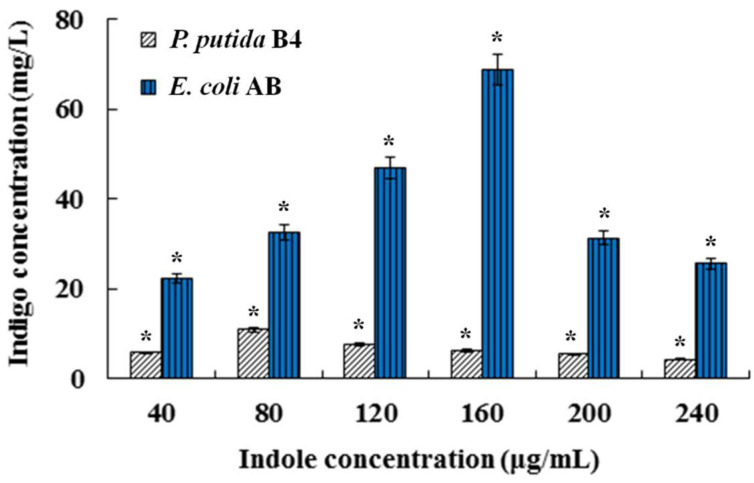
Indigo biosynthesis from different concentrations of indole in fermentation of *P. putida* B4 and *E. coli* AB. Bars with asterisk (*) are significantly different (*p* < 0.05).

The 1815 bp *styAB* gene was cloned from *P. putida* B4, and sequencing analysis showed that the DNA fragment shared a 100% homology with the styrene monooxygenase gene (GenBank accession no. DQ177365.1). The 6.3 kb recombinant expression vector pBK-AB was then constructed by inserting the *styAB* gene into pBK-CMV and transformed into *E. coli* DH5α, generating the recombinant strain *E. coli* AB. The fermentation results indicated that *E. coli* AB obtained the ability of indigo biosynthesis from indole, and its indigo production yield was quite higher than that of *P. putida* B4 (Figure 1). It was observed that indigo production in *E. coli* AB was indole dose-dependent. The highest yield of indigo (70 mg/L) in *E. coli* AB was produced from indole at 160 μg/mL, but higher concentrations of indole generated cytotoxicity and consequently led to a sharp decrease in indigo production (Figure 1).

**Figure 2:**
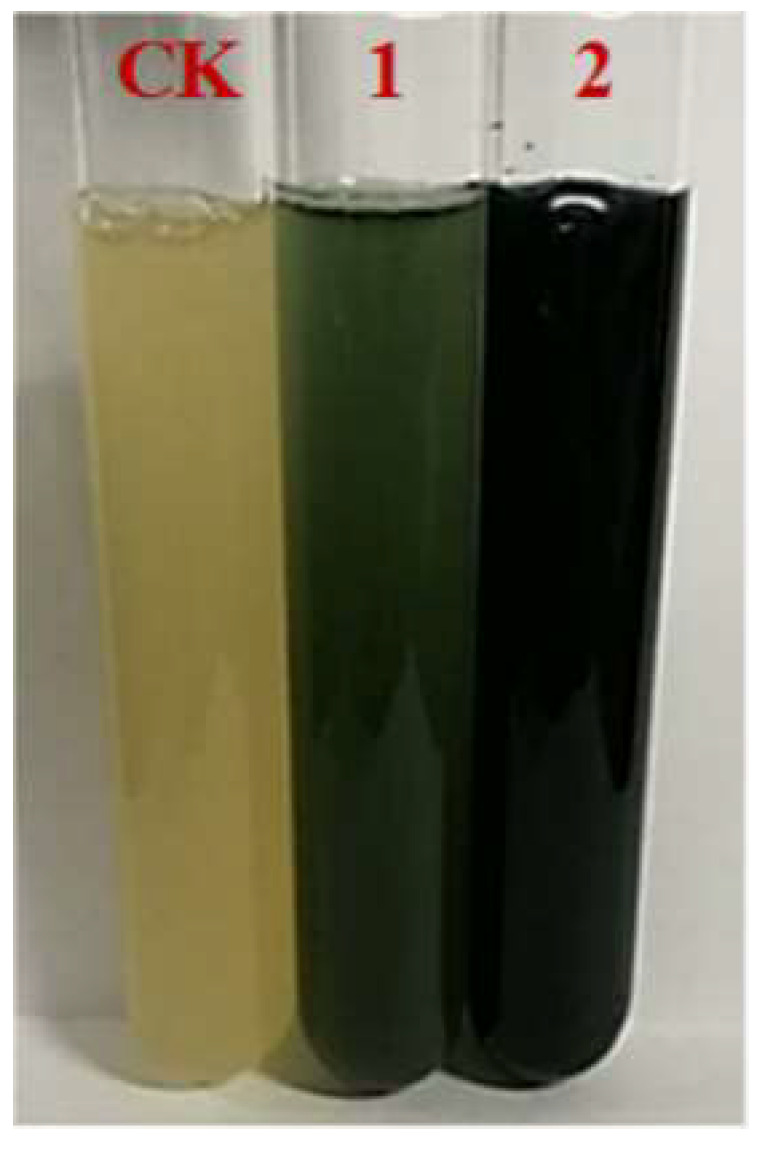
Indigo production from indole and tryptophan in *E. coli* AB fermentation. CK, *E. coli* DH5α; 1, *E. coli* AB fermentation with 160 μg/mL of indole; 2, *E. coli* AB fermentation with 1.0 mg/mL of tryptophan.

**Figure 3:**
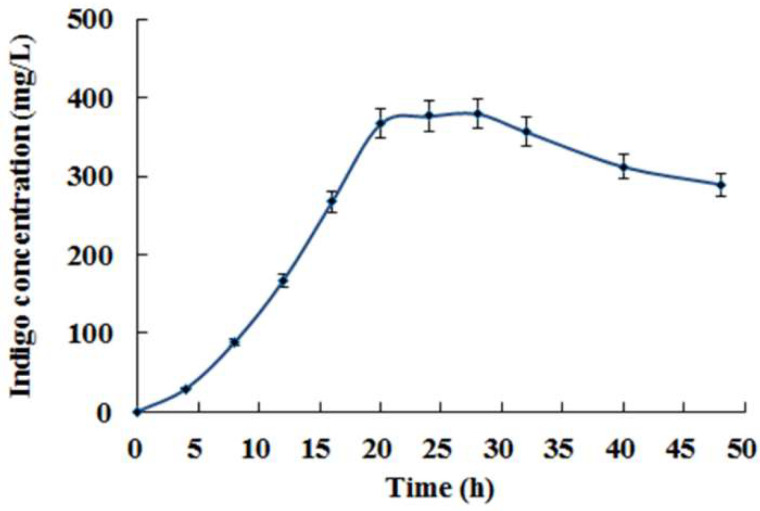
Indigo production from 1.0 mg/mL of tryptophan in *E. coli* AB fermentation.

In order to avoid the cytotoxicity of indole, tryptophan was used as the substrate of indigo biosynthesis. As shown in Figure 2, much more indigo was produced from tryptophan than that from indole in *E. coli* AB. The maximum yield of indigo from 1.0 mg/mL of tryptophan in *E. coli* AB was determined to be about 380 mg/L after 24 h of fermentation (Figure 3), which was about 5.4-fold higher than the highest yield (70 mg/L) from 160 μg/mL of indole, revealing that tryptophan was more suitable than indole as the substrate for indigo production by *E. coli* fermentation.

**Figure 4:**
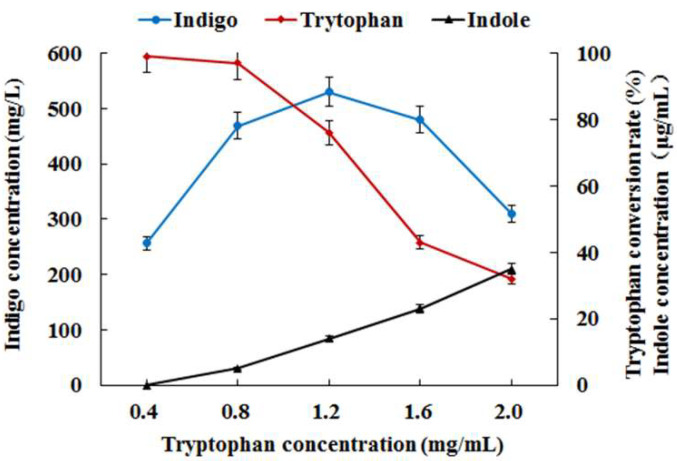
Indigo biosynthesis from different concentrations of tryptophan in *E. coli* AB fermentation.

Furthermore, the influence of different concentrations of tryptophan on indigo production was investigated in *E. coli* AB fermentation. The results (Figure 4) indicated that low concentrations of tryptophan (<0.8 mg/mL) could be almost completely transformed to indigo, but indigo yield was limited to some extent due to low substrate concentration. When the concentration of tryptophan increased to 0.8–1.2 mg/mL, though the conversion rate of tryptophan decreased to 75%, the maximum indigo yield was 530 mg/L from 1.2 mg/mL of tryptophan (Figure 4). However, as the concentration of tryptophan rose (>1.2 mg/mL), the conversion rate of tryptophan and indigo yield both fell rapidly. It suggested that excessive tryptophan possibly led to the inadequate catalytic capacity of tryptophanase for subsequent indigo biosynthesis.

### 3.2. Co-Expression of Monooxygenase Gene StyAB and Tryptophanase Gene TnaA for Indigo Biosynthesis in E. coli

**Figure 5:**
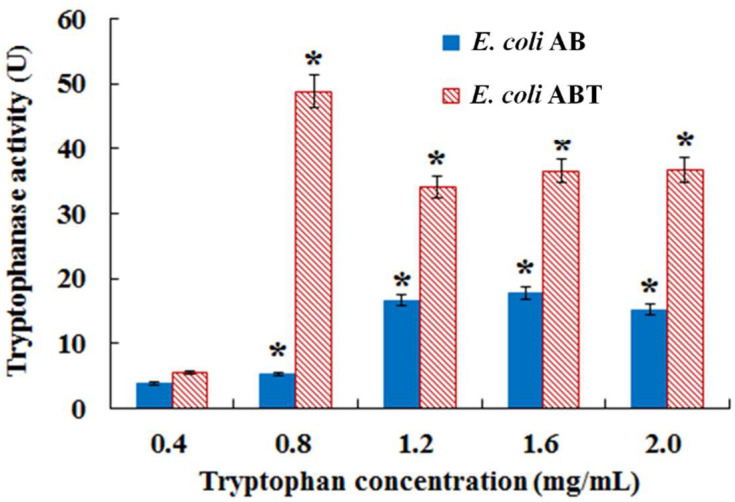
Tryptophanase activity in *E. coli* AB and *E. coli* ABT in fermentation with different concentrations of tryptophan. Bars with asterisk (*) are significantly different (*p* < 0.05).

In order to enhance the utilization rate of tryptophan and improve indigo biosynthesis, the tryptophanase gene *TnaA* was over-expressed in *E. coli* AB. The 1416 bp *TnaA* gene was cloned from *E. coli* DH5α, which shared a 100% homology with the *E. coli* tryptophanase gene (GenBank accession no. K00032.1). The 7.7 kb recombinant expression vector pBK-ABT was constructed by inserting the *TnaA* gene into pBK-AB and transformed into *E. coli* DH5α, generating the recombinant strain *E. coli* ABT. Tryptophanase activity assay indicated that the recombinant strain *E. coli* ABT with expression of *TnaA* exhibited much higher tryptophanase activity than *E. coli* AB in response to high concentrations (0.8–2.0 mg/mL) of tryptophan added in fermentation (Figure 5), demonstrating that the *TnaA* gene was successfully expressed in *E. coli* ABT.

**Figure 6:**
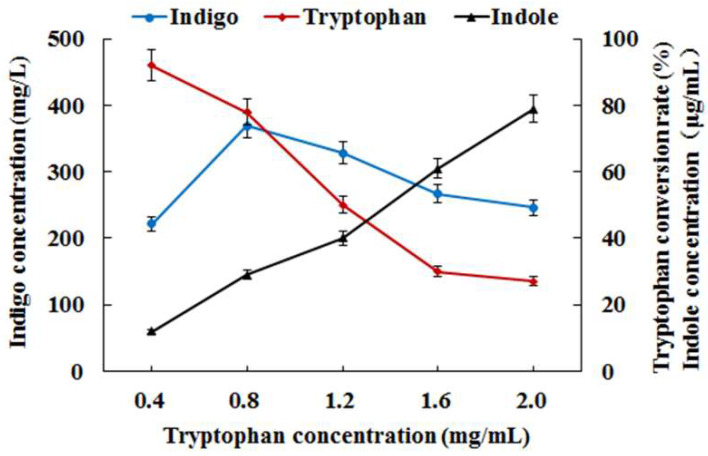
Indigo biosynthesis from different concentrations of tryptophan in *E. coli* ABT fermentation.

*E. coli* ABT was then used in fermentation with addition of different concentrations of tryptophan for indigo biosynthesis. Surprisingly, the fermentation results indicated that both the indigo yield and the conversion rate of tryptophan in *E. coli* ABT were significantly lower than that in *E. coli* AB at each concentration of tryptophan (Figure 6), revealing that expression of the *TnaA* gene hardly contributed to more indigo biosynthesis. Besides, more indole accumulated in *E. coli* ABT than that in *E. coli* AB, which was in accordance with the lower production yield of indigo (Figure 6). Though the tryptophanase activity was substantially enhanced, and tryptophan could be efficiently utilized, the monooxygenase activity must be strong enough for transformation of indole derived from tryptophan into indigo in *E. coli* ABT. It suggested that co-expression of the genes *TnaA* and *styAB* led to the deficiency in the catalytic activity of the monooxygenase StyAB because of some specific reasons, and it revealed that a delicate balance between the activities of tryptophanase and monooxygenase was essential for highly effective indigo biosynthesis from tryptophan in *E. coli*.

### 3.3. Introduction of Molecular Chaperone Enhanced the Activity of Monooxygenase StyAB and Indigo Biosynthesis in E. coli

**Figure 7:**
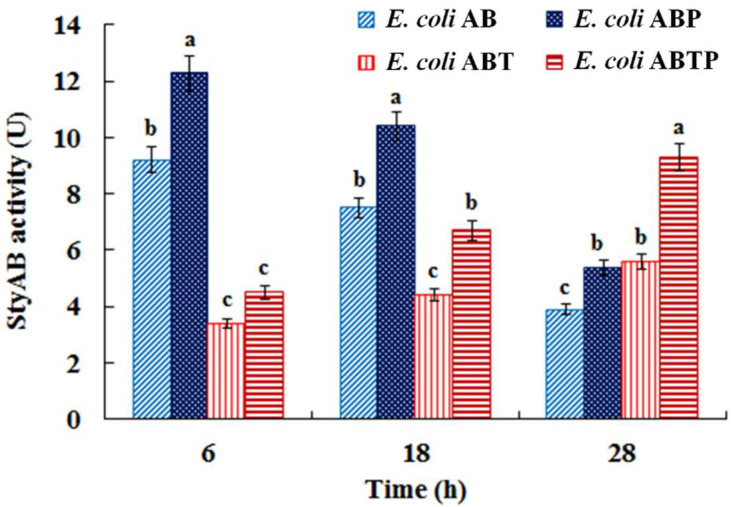
StyAB activity in *E. coli* AB, *E. coli* ABP, *E. coli* ABT, and *E. coli* ABTP in fermentation with 1.2 mg/mL of tryptophan. Bars with different letters (a, b, and c) are significantly different (*p* < 0.05).

In order to further enhance the activity of the monooxygenase StyAB, the molecular chaperone plasmid pGro7 was introduced into *E. coli* AB and *E. coli* ABT, respectively, generating the recombinant strain *E. coli* ABP and *E. coli* ABTP. Monooxygenase activity assay indicated that activities of the monooxygenase StyAB were significantly higher in the strain harboring pGro7 (*E. coli* ABP or *E. coli* ABTP) than in its corresponding strain without pGro7 (*E. coli* AB or *E. coli* ABT) during fermentation (Figure 7), which demonstrated that the presence of the molecular chaperone plasmid pGro7 contributed to improving the enzymatic activity in *E. coli*.

Moreover, it was observed that the strain with over-expression of *TnaA* (*E. coli* ABT or *E. coli* ABTP) expressed a significantly lower monooxygenase activity than its corresponding strain without *TnaA* expression (*E. coli* AB or *E. coli* ABP) when indigo was produced in quantity during fermentation (6 h and 18 h) (Figure 7), suggesting that expression of the *styAB* gene was reduced when it was co-expressed with the *TnaA* gene in the same vector.

**Figure 8:**
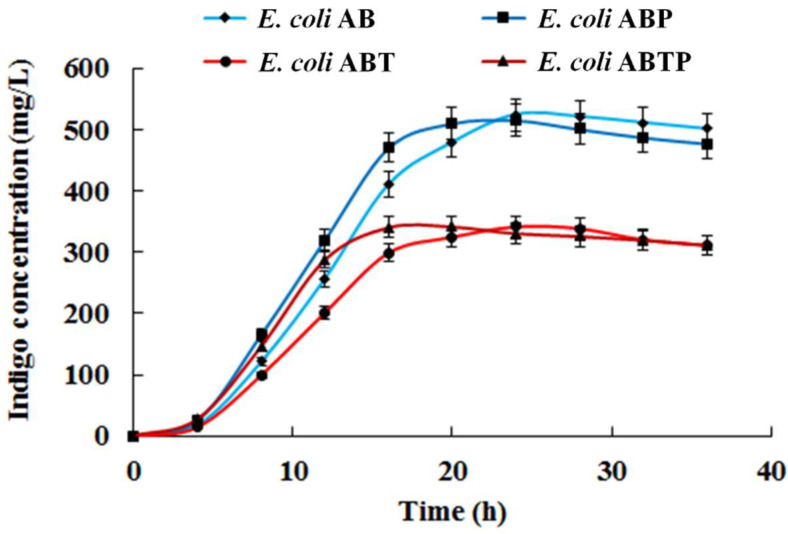
Indigo production from 1.2 mg/mL of tryptophan in fermentation of *E. coli* AB, *E. coli* ABP, *E. coli* ABT, and *E. coli* ABTP.

Fermentation results showed that indigo accumulated faster in the first 24 h of fermentation in strains *E. coli* ABP and *E. coli* ABTP than in *E. coli* AB and *E. coli* ABT (Figure 8). Though a significant difference in the maximum yield of indigo was hardly detected between strains with and without pGro7, the introduction of the molecular chaperone benefited the catalytic activity of StyAB and consequently led to more indigo production (Figure 8). Besides, in comparison with *E. coli* ABT and *E. coli* ABTP, about 2-fold higher maximum yield of indigo was achieved in *E. coli* AB and *E. coli* ABP after 20 h of fermentation (Figure 8), which demonstrated that co-expression of *TnaA* with *styAB* exerted a negative effect on indigo biosynthesis due to a great reduction in the monooxygenase StyAB activity.

**Figure 9:**
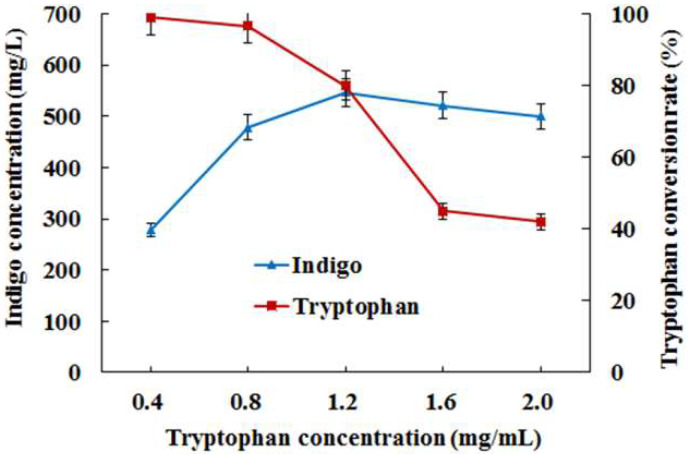
Indigo biosynthesis from different concentrations of tryptophan in *E. coli* ABP fermentation.

Furthermore, the fermentation with different concentrations of tryptophan showed that the conversion rate of tryptophan declined as the concentration of tryptophan rose, and the maximum yield of indigo was detected to be 550 mg/L from 1.2 mg/mL of tryptophan in fermentation of *E. coli* ABP (Figure 9).

## 4. Discussion

Different strategies of genetic manipulations were conducted to enhance indigo biosynthesis in *E. coli* in this study. The styrene monooxygenase gene *styAB* from *P. putida* was successfully expressed in *E. coli,* and it fulfilled a large number of indigo biosynthesis, demonstrating that the monooxygenase StyAB was the key enzyme for indigo biosynthesis. In *P. putida*, both styrene monooxygenase and styrene oxide isomerase are indispensable for transformation of indole to indigo [9]. The conversion of indole to indigo in *P. putida* is the result of a two-step biotransformation including formation of indole oxide from indole by styrene monooxygenase and generation of 3-oxindole from indole oxide by styrene oxide isomerase, and indigo is finally synthesized by dimerization of 3-oxindole [18]. This study verified that indigo production from indole could be simply achieved by expression of solo styrene monooxygenase in *E. coli*, which is an economic pathway for industrial production. Further, it suggested that there was a possible alternative isoenzyme of styrene oxide isomerase that could catalyze indole oxide to 3-oxindole in *E. coli*.

**Figure 10:**
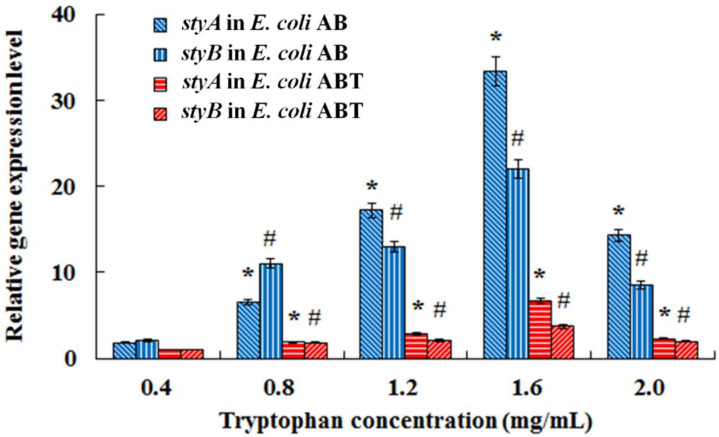
Transcriptional level assay of the genes *styA* and *styB* in *E. coli* AB and *E. coli* ABT in fermentation with different concentrations of tryptophan. Bars with asterisk (*) or pound sign (#) are significantly different (*p* < 0.05).

The monooxygenase StyAB directly catalyzes indole into indigo, but a high concentration of indole was toxic to *E. coli* cells [19,20], so there are inevitable limitations for indigo production using indole as the substrate. As *E. coli* could transform tryptophan to indole by catalysis of tryptophanase [21], tryptophan was used as the alternative substrate in fermentation for indigo production. The tryptophanase gene *TnaA* was successfully co-expressed with the monooxygenase gene *styAB* in *E. coli*, which obviously enhanced the tryptophanase activity. However, expression of *TnaA* unexpectedly resulted in a decrease in the monooxygenase activity, and consequently, co-expression of *TnaA* with *styAB* failed to promote indigo biosynthesis from tryptophan. In order to figure out the reason for StyAB activity decrease, the transcriptional level assay of the genes *styA* and *styB* was performed in *E. coli* AB and *E. coli* ABT in fermentation with different concentrations of tryptophan. As shown in Figure 10, the relative expression levels of both *styA* and *styB* in *E. coli* AB were significantly higher than that in *E. coli* ABT, which demonstrated that the StyAB activity decrease resulted from the low expression of *styAB* in *E. coli* ABT. For construction of the co-expression vector pBK-ABT, the gene *TnaA* was inserted into the upstream region of *styAB*. *TnaA* was adjacent to the promoter and consequently kept *styAB* distant from the promoter region. As the two genes were under the control of the same promoter, the transcription of *styAB* was probably attenuated due to the longer distance from the promoter [22]. Royo et al. [23] reported that co-expression of tryptophanase from *E. coli* and dioxygenase from *Sphingomonas macrogolitabida* did not improve the rate of indigo production from tryptophan in *E. coli*, and the reason probably was that tryptophanase production is not a limiting factor. Indigo biosynthesis from tryptophan in *E. coli* is a cascade reaction involved with sequential catalysis of tryptophanase and monooxygenase, so it is reasonable to assume that an appropriate balance in activities of the two key enzymes is likely essential for achievement of highly efficient indigo production. Though the genes *TnaA* and *styAB* were co-expressed in *E. coli*, their expressions were not under a tightly regulatory control. Hence, it was possible that the irrational ratio of expression levels of *TnaA* and *StyAB* led to indole accumulation and indigo biosynthesis decline.

The plasmid pGro7 harboring the chaperone protein groES-groEL could help the recombinant protein fold correctly in *E. coli* [24]. Therefore, introduction of pGro7 significantly enhanced the activity of StyAB and consequently sped up the rate of indigo biosynthesis from tryptophan. However, the effect of the molecular chaperone on the production yield of indigo was very finite. It suggested that other limiting factors might exit in the indigo biosynthesis system. Efficient cofactor regeneration is critical to oxidation-reduction reactions. Since the epoxidation of indole requires the activity of the flavin-dependent monooxygenase StyAB, enough supply of NADH or FAD is essential for the reaction system and productivity of indigo biosynthesis [25]. It is expectable that introduction of a NADH or FAD regeneration system will further promote indigo production by catalysis of the monooxygenase StyAB in *E. coli*.

## 5. Conclusions

The styrene monooxygenase gene *styAB* from *P. putida* was heterologously over-expressed in *E. coli*, and it fulfilled an economic pathway for indigo production from indole and tryptophan. Introduction of the chaperone protein groES-groEL significantly enhanced the catalytic activity of StyAB and consequently sped up the rate of indigo biosynthesis from tryptophan.

## Figures and Tables

**Table 1:** Strains and plasmids used in this work.

Strains or Plasmids	Relevant Features	Source
Plasmids		
pBK-CMV	Vector for gene expression in *E. coli*, Kan^R^	Laboratory collection
pBK-AB	*styAB* gene cloned in pBK-CMV, Kan^R^	This work
pBK-ABT	*TnaA* gene cloned in pBK-AB, Kan^R^	This work
pGro7	Chaperone plasmid harboring *groES-groEL*, Cm^R^	Takara, Beijing, China
Strains		
*E. coli* DH5α	Host for gene expression	TIANGEN, Beijing, China
*E. coli* AB	*E. coli* DH5α harboring pBK-AB	This work
*E. coli* ABT	*E. coli* DH5α harboring pBK-ABT	This work
*E. coli* ABTP	*E. coli* DH5α harboring pBK-ABT and pGro7	This work
*P. putida* B4	Donor of *styAB* gene	Laboratory collection

**Table 2:** Primers used in this work.

Gene	Primer	Sequence (5′–3′)
	q RT PCR	
*styA*	styA-F	GGCGAGCTGATTGAGATTC
	styA-R	TTTTGCCGTTATTGAGGGT
*styB*	styB-F	AAAAGATGTGGTGGTGGAT
	styB-R	TGCTGAAGAATGCCGATAA
16S rRNA	16S-F	CCACCTGGACTGATACT
	16S-R	GCACCTGTCTCAATGTT
	PCR	
*styAB*	styAB-F	AACTGCAGATGAAAAAGCGTATCGGTATTG
	styAB-R	CCCAAGCTTTCAATTCAGTGGCAACGGGTT
*TnaA*	TnaA-F	CCCAAGCTTATGGAAAACTTTAAACATCTCCC
	TnaA-R	CTAGTCTAGATTAAACTTCTTTAAGTTTTGCGG

## Data Availability

Data is contained within the article.

## Supplementary Materials

The following supporting information can be downloaded at: https://www.mdpi.com/article/10.3390/foods11142117/s1, Figure S1: Construction of the expression vector pBK-AB harboring *styAB* and pBK-ABT harboring *TnaA* and *styAB*.

## References

[1] Erkan G., Şengül K., Kaya S. (2014). Dyeing of white and indigo dyed cotton fabrics with *Mimosa tenuiflora* extract. J. Saudi Chem. Soc..

[2] Han G.H., Bang S.E., Babu B.K., Chang M., Shin H.-J., Kim S.W. (2011). Bio-indigo production in two different fermentation systems using recombinant Escherichia coli cells harboring a flavin-containing monooxygenase gene (*fmo*). Process Biochem..

[3] Bechtold T., Turcanu A., Geissler S., Ganglberger E. (2002). Process balance and product quality in the production of natural indigo from Polygonum tinctorium Ait. applying low-technology methods. Bioresour. Technol..

[4] Pathak H., Madamwar D. (2010). Biosynthesis of indigo dye by newly isolated naphthalene-degrading strain *Pseudomonas* sp. HOB1 and its application in dyeing cotton fabric. Appl. Biochem. Biotechnol..

[5] Bhushan B., Samanta S.K., Jain R.K. (2000). Indigo production by naphthalene-degrading bacteria. Lett. Appl. Microbiol..

[6] Doukyu N., Nakano T., Okuyama Y., Aono R. (2002). Isolation of an *Acinetobacter* sp. ST-550 which produces a high level of indigo in a water-organic solvent two-phase system containing high levels of indole. Appl. Microbiol. Biotechnol..

[7] Gillam E.M., Aguinaldo A.M., Notley L.M., Kim D., Mundkowski R.G., Volkov A.A., Arnold F.H., Soucek P., DeVoss J.J., Guengerich F.P. (1999). Formation of indigo by recombinant mammalian cytochrome P450. Biochem. Biophys. Res. Commun..

[8] Mercadal J.P., Isaac P., Sineriz F., Ferrero M.A. (2010). Indigo production by *Pseudomonas* sp. J26, a marine naphthalene-degrading strain. J. Basic Microbiol..

[9] O’Connor K.E., Dobson A.D., Hartmans S. (1997). Indigo formation by microorganisms expressing styrene monooxygenase activity. Appl. Environ. Microbiol..

[10] Lin G.H., Chen H.P., Huang J.H., Liu T.T., Lin T.K., Wang S.J., Tseng C.H., Shu H.Y. (2012). Identification and characterization of an indigo-producing oxygenase involved in indole 3-acetic acid utilization by *Acinetobacter baumannii*. Antonie Van Leeuwenhoek.

[11] Qu Y., Ma Q., Zhang X., Zhou H., Li X., Zhou J. (2012). Optimization of indigo production by a newly isolated *Pseudomonas* sp. QM. J. Basic Microbiol..

[12] Mermod N., Harayama S., Timmis K.N. (1986). New route to bacterial production of indigo. Nat. Biotechnol..

[13] Furuya T., Takahashi S., Ishii Y., Kino K., Kirimura K. (2004). Cloning of a gene encoding flavin reductase coupling with dibenzothiophene monooxygenase through coexpression screening using indigo production as selective indication. Biochem. Biophys. Res. Commun..

[14] Qu Y., Shi S., Zhou H., Ma Q., Li X., Zhang X., Zhou J. (2012). Characterization of a novel phenol hydroxylase in indoles biotransformation from a strain *Arthrobacter* sp. W1. PLoS ONE.

[15] Cheng L., Yin S., Chen M., Sun B., Hao S., Wang C. (2016). Enhancing indigo production by over-expression of the styrene monooxygenase in *Pseudomonas putida*. Curr. Microbiol..

[16] Green M., Sambrook J. (2012). Molecular Cloning: A Laboratory Manual.

[17] Schmittgen T.D., Livak K.J. (2008). Analyzing real-time PCR data by the comparative C_T_ method. Nat. Protoc..

[18] O’Leary N.D., O’Connor K.E., Dobson A.D. (2002). Biochemistry, genetics and physiology of microbial styrene degradation. FEMS Microbiol. Rev..

[19] Garbe T.R., Kobayashi M., Yukawa H. (2000). Indole-inducible proteins in bacteria suggest membrane and oxidant toxicity. Arch. Microbiol..

[20] Wang D., Ding X., Rather P.N. (2001). Indole can act as an extracellular signal in *Escherichia coli*. J. Bacteriol..

[21] Snell E.E. (1975). Tryptophanase: Structure, catalytic activities, and mechanism of action. Adv. Enzymol. Relat. Areas Mol. Biol..

[22] Rydenfelt M., Garcia H.G., Cox R.S., Phillips R. (2014). The influence of promoter architectures and regulatory motifs on gene expression in *Escherichia coli*. PLoS ONE.

[23] Royo J.L., Moreno-Ruiz E., Cebolla A., Santero E. (2005). Stable long-term indigo production by overexpression of dioxygenase genes using a chromosomal integrated cascade expression circuit. J. Biotechnol..

[24] Nishihara K., Kanemori M., Kitagawa M., Yanagi H., Yura T. (1998). Chaperone coexpression plasmids: Differential and synergistic roles of DnaK-DnaJ-GrpE and GroEL-GroES in assisting folding of an allergen of Japanese cedar pollen, Cryj2, in *Escherichia coli*. Appl. Environ. Microbiol..

[25] Otto K., Hofstetter K., Röthlisberger M., Witholt B., Schmid A. (2004). Biochemical characterization of StyAB from *Pseudomonas* sp. strain VLB120 as a two-component flavin-diffusible monooxygenase. J. Bacteriol..

