# Supplementary material for: Production of Indigo by Recombinant Escherichia coli with Expression of Monooxygenase, Tryptophanase, and Molecular Chaperone
Source: Foods. 2022 Jul 16;11(14):2117. doi: 10.3390/foods11142117 (PMC9320885; doi:10.3390/foods11142117)
Supplement: Supplementary file 1 [file foods-11-02117-s001.zip › foods-1788992-supplementary.pdf]

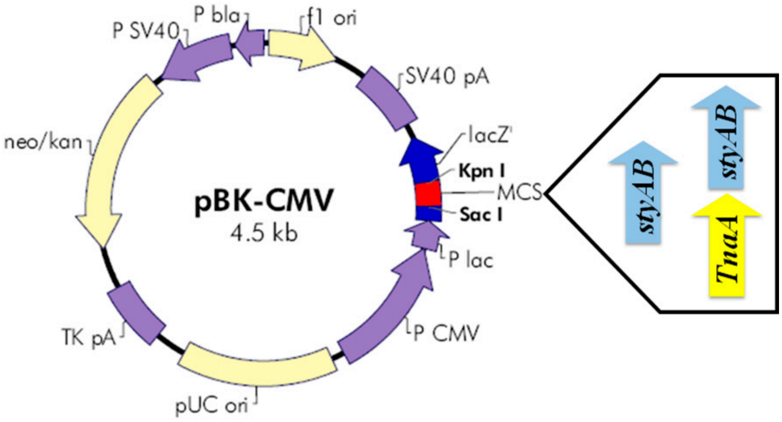

Figure S1. Construction of the expression vector pBK-AB harboring *styAB* and pBK-ABT harboring *TnaA* and *styAB*. MCS, multiple cloning site; Plac, lac promoter; PCMV, CMV promoter; pUC ori, pUC origin; neo/kan, neomycin/kanamycin resistance ORF.
